# Supplementary figures and images for: Brain activations during execution and observation of visually guided sequential manual movements in autism and in typical development: A study protocol
Source: PLoS One. 2024 Jun 24;19(6):e0296225. doi: 10.1371/journal.pone.0296225 (PMC11195952; doi:10.1371/journal.pone.0296225)

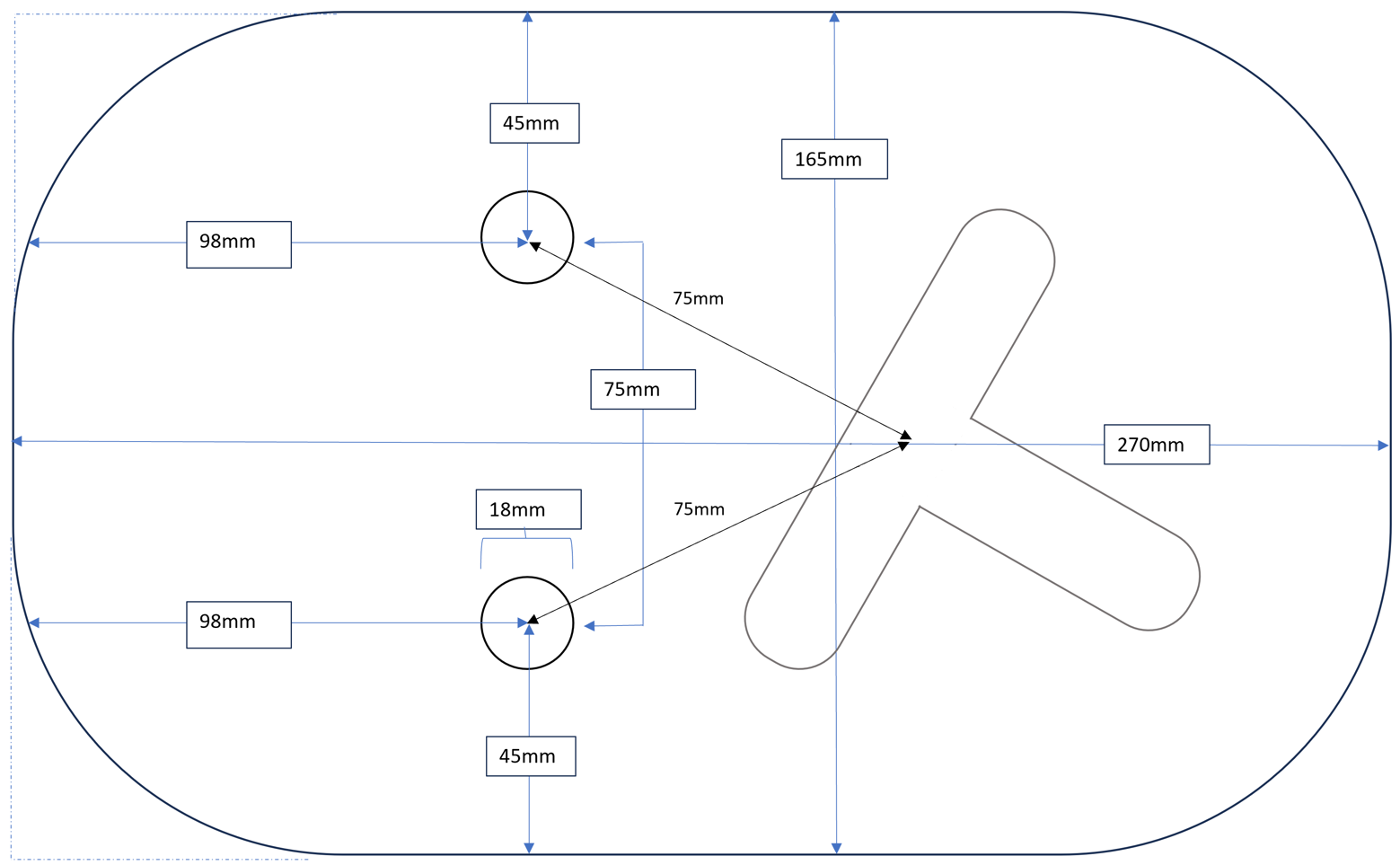

Supplement: S1 Fig — (TIF) [file pone.0296225.s001.tif]

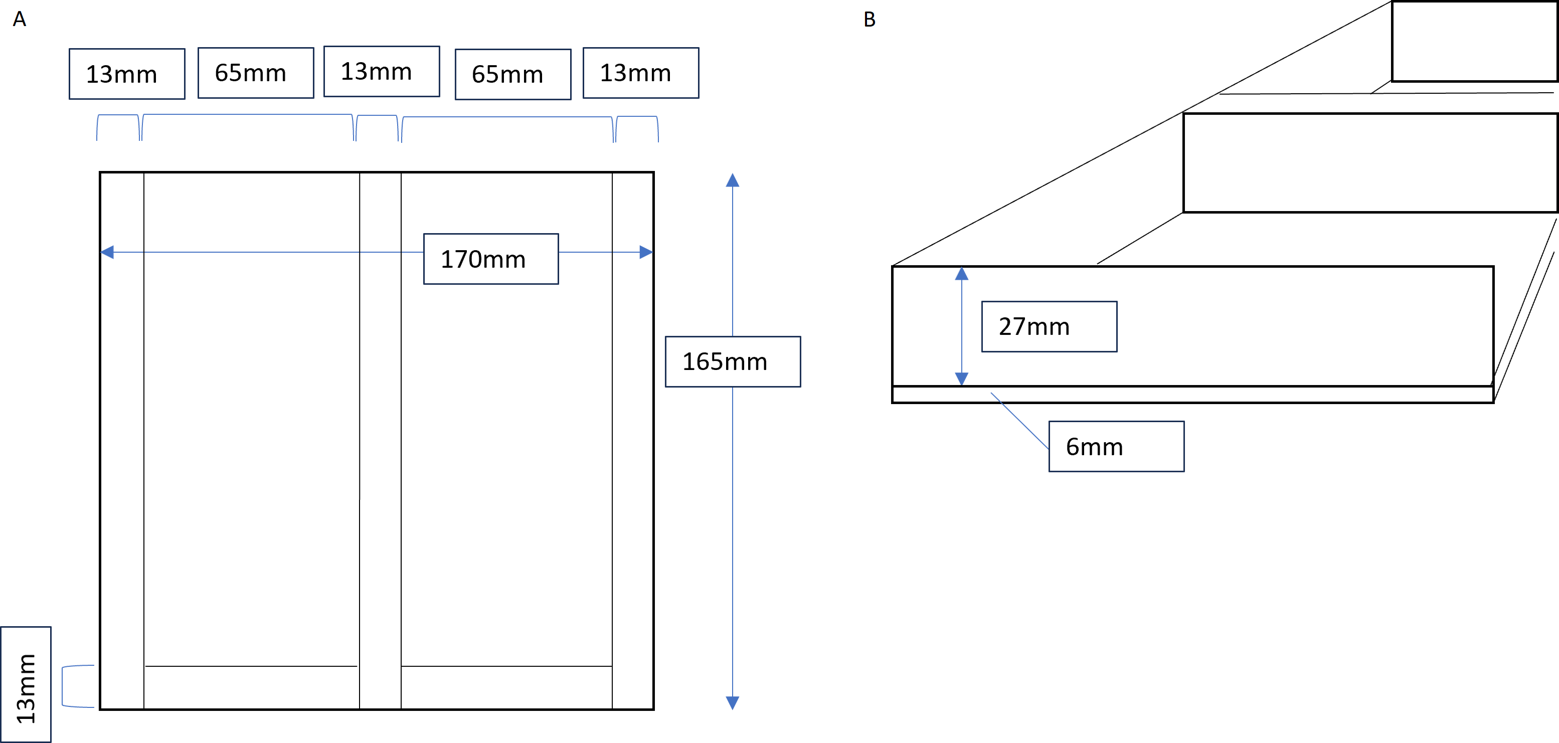

Supplement: S2 Fig — (A) Box with compartments (1:2 scale), and (B) 3D drawing including measures of box base plate and side heights (not to scale). (TIF) [file pone.0296225.s002.tif]

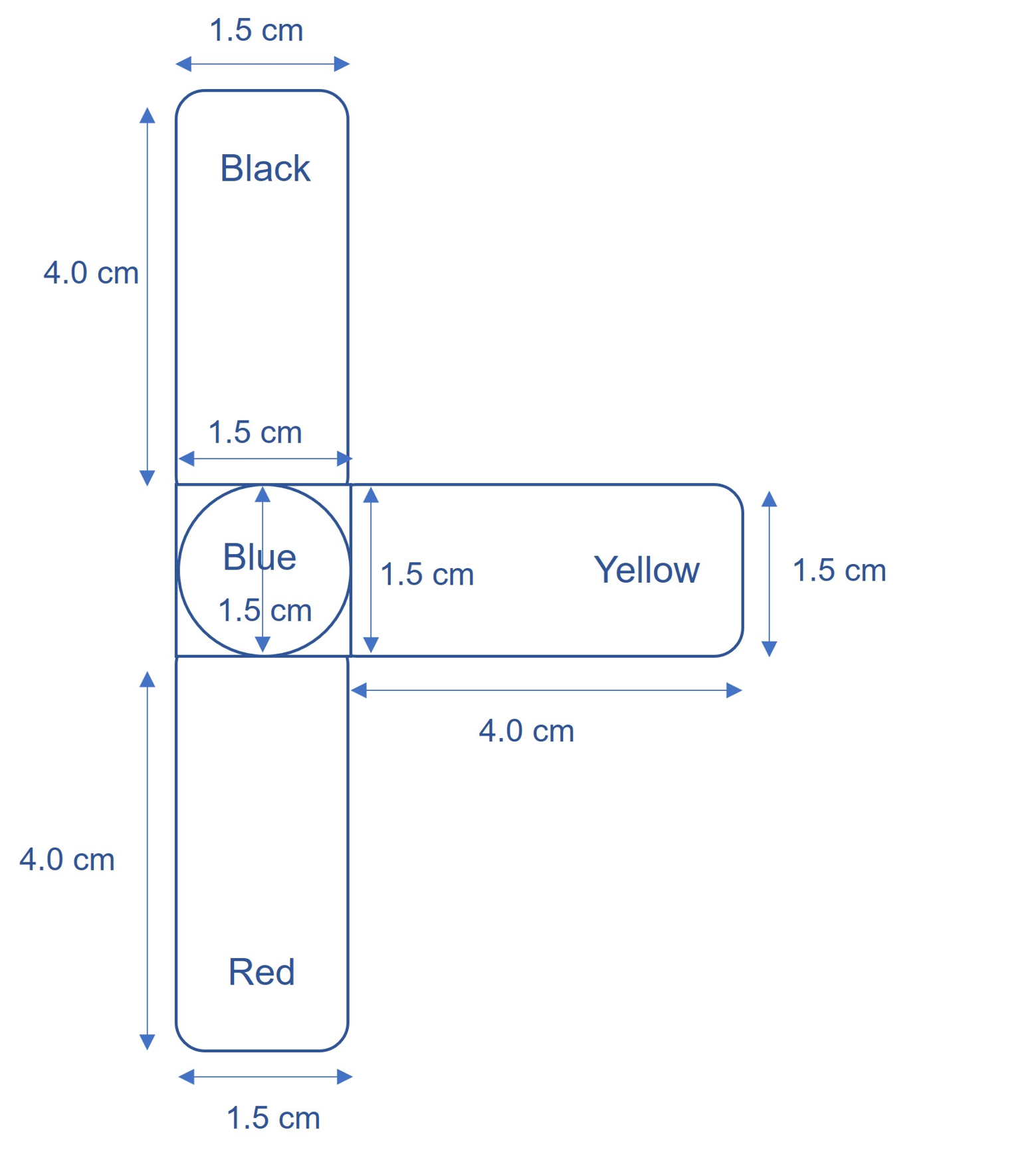

Supplement: S3 Fig — (TIF) [file pone.0296225.s003.tif]
