# Supplementary material for: Brain activations during execution and observation of visually guided sequential manual movements in autism and in typical development: A study protocol
Source: PLoS One. 2024 Jun 24;19(6):e0296225. doi: 10.1371/journal.pone.0296225 (PMC11195952; doi:10.1371/journal.pone.0296225)
Supplement: S1 File — (DOCX) [file pone.0296225.s008.docx]

**English translation of ethics approval document**

**DECISION**

2022-05-25

**Applicant Research Principal**

Umeå University

**Researcher carrying out the project**

Erik Domellöf

**Project title**

Motor planning in children/young people with autism spectrum disorder: what happens in the brain?

**Information about the application**

The application was received by the Ethics Review Authority on 2022-03-15 and became valid on 2022-03-18. The application is previously processed at the meeting on 04/06/2022. Completion requested by the authority according to decision was received 2022-05-03.

The ethics review authority decides as follows.

**DECISION**

The Ethics Review Authority approves the research specified in the application, with the following condition:

For children 12-14 years of age, there should not be a consent form as they cannot themselves consent to participation.
