# Supplementary material for: Brain activations during execution and observation of visually guided sequential manual movements in autism and in typical development: A study protocol
Source: PLoS One. 2024 Jun 24;19(6):e0296225. doi: 10.1371/journal.pone.0296225 (PMC11195952; doi:10.1371/journal.pone.0296225)
Supplement: S2 File — (PDF) [file pone.0296225.s009.pdf]

BESLUT

2022-05-25

Sökande forskningshuvudman
Umeå universitet

Forskare som genomför projektet
Erik Domellöf

Projekttitel
Motorisk planering hos barn/unga med autismspektrumtillstånd: vad händer i hjärnan?

Uppgifter om ansökan
Ansökan inkom till Etikprövningsmyndigheten 2022-03-15 och blev valid 2022-03-18. Ansökan är tidigare behandlad vid sammanträde 2022-04-06. Av myndigheten begärd komplettering enligt beslut inkom 2022-05-03.

Etikprövningsmyndigheten beslutar enligt nedan.

BESLUT

Etikprövningsmyndigheten godkänner den forskning som anges i ansökan, med följande villkor:

För barn 12-14 år ska det inte finnas en samtyckesblankett då dessa inte själva kan samtycka till deltagande.

Det här beslutet kan överklagas hos Överklagandenämnden för etikprövning. Hur man överklagar framgår av bifogad anvisning.

På Etikprövningsmyndighetens vägnar

Katja Isberg Amnäs
Ordförande

Beslutet har fattats av följande personer:

Ordförande
Katja Isberg Amnäs

Beslutet har fattats efter föredragning av vetenskaplig sekreterare
Erik Näslund

Beslutet sänds till

Ansvarig forskare: Erik Domellöf

Forskningshuvudmannens företrädare: Annika Nordlund

Hur man överklagar Etikprövningsmyndighetens beslut

Vem får överklaga?

Det är forskningshuvudmannen som får överklaga Etikprövningsmyndighetens beslut om det har gått sökanden emot. Överklagandet ska vara skriftligt. Skrivelsen ska vara undertecknad av behörig företrädare för forskningshuvudmannen.

Om forskaren överklagar ska en fullmakt från forskningshuvudmannen bifogas.

När ska beslutet senast överklagas?

Överklagandet ska ha kommit in till Etikprövningsmyndigheten inom tre veckor från den dag då forskningshuvudmannen fick del av beslutet.

Vad ska överklagandet innehålla?

Överklagandet ska innehålla uppgifter om

1. klagandens namn, person- eller organisationsnummer, adress, telefonnummer och e-postadress
2. det beslut som överklagas (dag för beslut, projekttitel och diarienummer)
3. hur ni anser att myndighetens beslut ska ändras och skälen till att beslutet bör ändras.

Var ska överklagandet skickas?

Överklagandet ska ställas till Överklagandenämnden för etikprövning. Men det ska skickas eller lämnas till Etikprövningsmyndigheten.

Om överklagandet har kommit in i rätt tid överlämnar myndigheten överklagandet och handlingarna till Överklagandenämnden för etikprövning.
